# Supplementary material for: Chromothripsis during telomere crisis is independent of NHEJ, and consistent with a replicative origin
Source: Genome Res. 2019 May;29(5):737–49. doi: 10.1101/gr.240705.118 (PMC6499312; doi:10.1101/gr.240705.118)
Supplement: Supplemental Material [file supp_gr.240705.118_Supplemental_file_1.zip › contigs/annotated_contigs/DB99/contig.2.DB99_length_458_mean_cov_8.47816593886.docx]

**DB99_length_458_mean_cov_8.47816593886**

```
TGGGTTCAGACGTGTGCTCTTCCGATCT|AAAATAGGAAGAGAGTTTGCAAAATAGGAGCATTTACCAGAAGCCTCTCT|CTCCCTCCC
                             >chr8:144119973-144120060 - E=9e-35 p=0e+00       >chr8:14
TCCCTCCCTCCCTCTCTCTCTCTCTC|TGTGTCTCTCAAGTAGAAGTGGGGTCTCTATGTTGCCCAGGCTGCTCTTGAACTCCGAGGCT
4119460-144119840 - E=2e-208
CAAGCGATCCTCCTGCCTCAGCCTCCCAAAGTGCTGCGATTACAGGTGTGAACTACTGGGCCCAGCCCCAGGCCCTCTTACAGGAGTGG

GCTGCCTAGAAACATTCCAGTGCCATATCCCACTCCACTCCGGGTGCAGGGACAGGACCCCTGCCACCCCCAACCCAGGCCCAAAAGGA

GGGGCCAGGAGGGGAGGGGCCACCAAGGCAGGTGAGCTGCTTAAGAGGAGCGAGGGCTGTGGACAGTCCTGCGGACTGCAGGAGGGGCC

AGCAGGATCAATATCC
```
